# Nasal application of neuropeptide S inhibits arthritis pain-related behaviors through an action in the amygdala

**DOI:** 10.1186/1744-8069-10-32

**Published:** 2014-05-29

**Authors:** Georgina Medina, Guangchen Ji, Stéphanie Grégoire, Volker Neugebauer

**Affiliations:** 1 Department of Neuroscience and Cell Biology, University of Texas Medical Branch at Galveston, 301 Univ. Blvd. RT1069, Galveston, TX 77555-1069, USA

**Keywords:** Pain, Amygdala, Neuropeptide S, Emotion, Anxiety, Brain, Sensitization

## Abstract

Recently discovered neuropeptide S (NPS) has anxiolytic and pain-inhibiting effects in rodents. We showed previously that NPS increases synaptic inhibition of amygdala output to inhibit pain behaviors. The amygdala plays a key role in emotional-affective aspects of pain. Of clinical significance is that NPS can be applied nasally to exert anxiolytic effects in rodents. This study tested the novel hypothesis that nasal application of NPS can inhibit pain-related behaviors in an arthritis model through NPS receptors (NPSR) in the amygdala. Behaviors and electrophysiological activity of amygdala neurons were measured in adult male Sprague Dawley rats. Nasal application of NPS, but not saline, inhibited audible and ultrasonic vocalizations and had anxiolytic-like effects in the elevated plus-maze test in arthritic rats (kaolin/carrageenan knee joint arthritis model) but had no effect in normal rats. Stereotaxic application of a selective non-peptide NPSR antagonist (SHA68) into the amygdala by microdialysis reversed the inhibitory effects of NPS. NPS had no effect on hindlimb withdrawal thresholds. We showed previously that intra-amygdala application of an NPSR antagonist alone had no effect. Nasal application of NPS or stereotaxic application of NPS into the amygdala by microdialysis inhibited background and evoked activity of amygdala neurons in arthritic, but not normal, anesthetized rats. The inhibitory effect was blocked by a selective NPSR antagonist ([D-Cys(tBu)^5^]NPS). In conclusion, nasal application of NPS can inhibit emotional-affective, but not sensory, pain-related behaviors through an action in the amygdala. The beneficial effects of non-invasive NPS application may suggest translational potential.

## Background

Pain is a multidimensional experience with sensory-discriminative, emotional-affective and cognitive aspects [1,2]. Clinical and pre-clinical work now recognizes the amygdala as an important neural substrate for the emotional-affective dimension of pain [3-6]. In animals, activity in the amygdala has been shown to correlate positively with pain behaviors, and interventions that deactivate the amygdala have inhibitory effects in different pain models [4,6-12]. In humans, increased amygdala activity has been found in experimental and clinical pain conditions [5,13-16]. The amygdala circuitry that contributes to the emotional-affective component of pain is centered on the lateral-basolateral (LA-BLA) and central (CeA) nuclei [3,4]. The CeA serves output functions whereas the LA-BLA network provides highly processed nociceptive and affected-related information to the CeA. Interposed between LA-BLA and CeA is a cluster of GABAergic intercalated cells (ITC). These dorsomedial ITC cells serve as a feedforward inhibitory gate to control amygdala output from the CeA, which is currently considered a key mechanism of behavioral extinction of negative emotions such as fear [17-22].

Importantly, there is evidence to suggest that neuropeptide S (NPS), a newly discovered 20 amino-acid peptide, selectively enhances dorsomedial ITC-dependent feedforward inhibition of CeA neurons to produce powerful anxiolytic effects [23]. NPS binds with high affinity to a Gq/Gs-coupled receptor (NPSR) to increase intracellular calcium and cAMP-PKA signaling [24,25]. NPSR mRNA is expressed in discrete brain areas including the rat amygdala where high levels of NPSR mRNA are found in the ITC but not LA, BLA and CeA [26] although NPSR protein appears to be absent in these areas under normal conditions [27]. We hypothesized that targeting ITC amygdala cells would also be a useful strategy to control pain-related amygdala activity and emotional-affective aspects of pain behaviors. Recent work from our group showed decreased feedforward inhibition of laterocapsular CeA neurons in an arthritis pain model [28,29]. Therefore, restoring inhibition of the CeA with NPS could be a useful pharmacological strategy.

Intracerebroventricular administration of NPS was shown to inhibit aversive and anxiety-like behaviors [23,30,31] and these effects were mimicked by injections of NPS directly into the amygdala [32,33]. Intracerebroventricular administration of NPS also had antinociceptive effects in the tail-flick, hot-pate and formalin tests [34,35], and we showed recently that direct application of NPS into the ITC inhibited emotional-affective responses in an arthritis pain model [29]. Importantly, recent evidence suggests that NPS can be administered nasally to exert anxiolytic effects [36,37]. The goal of the present study was to examine behavioral effects of NPS administered using a non-invasive (nasal) method; determine the contribution of NPSR in the ITC area of the amygdala to the effects of nasally applied NPS; and show inhibitory effects of NPS administered nasally or directly into the ITC on the activity of CeA output neurons. The results show that nasally applied NPS can inhibit emotional-affective behaviors in an arthritis pain model through an action in the amygdala without affecting sensory or baseline responses.

## Results

In the behavioral experiments, audible and ultrasonic vocalizations, anxiety-like behavior and spinal reflexes (hindlimb withdrawal thresholds) were measured in adult male Sprague Dawley rats with (n = 29 rats) or without (n = 12 rats) a kaolin/carrageenan-induced arthritis as described in Methods (Experimental Protocol). Electrophysiological recordings of amygdala neurons were also performed in adult male Sprague Dawley rats with (n = 20 rats) or without (n = 5 rats) arthritis (kaolin/carrageenan model). Normal animals without arthritis served as a control group. These animals did not receive any needle insertion or vehicle injection to avoid any latent confounding effect of injury and/or increased intraarticular pressure. Intraarticular saline injection causes a temporary swelling of the joint [38], which is one of the cardinal symptoms of an inflammation.

### Inhibitory effect of nasal NPS on vocalizations of arthritic rats

#### *Audible vocalizations*

**Figure 1:**
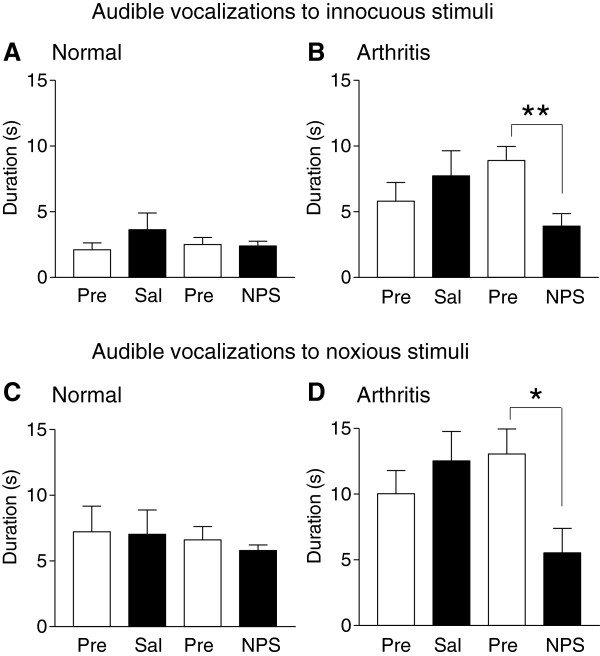
**NPS effects on audible vocalizations.** Audible vocalizations were evoked by brief (15 s) innocuous (300 g/30 mm^2^, **A**, **B**) and noxious (1200 g/30 mm^2^, **C**, **D**) stimulation of the knee joint in normal and arthritic rats. Duration of vocalizations during a 1 min period was measured before (Pre) and 45 min after nasal application of saline (Sal; n = 6 normal rats; n = 9 arthritis rats) or before (Pre) and 45 min after NPS (14 nmol; n = 6 normal rats; n = 8 arthritic rats). NPS had significant inhibitory effects in the arthritic pain model. *,** P < 0.05, 0.01, unpaired *t*-test.

Audible vocalizations evoked by brief (15 s) mechanical compression of the knee with innocuous (300 g/30 mm^2^) and noxious (1200 g/30 mm^2^) intensities were measured in normal (Figure 1A and C) and arthritic rats (Figure 1B and D). NPS or saline were applied topically onto the rhinarium (10 μl on each side) as described in the literature [36]. In normal rats, nasal application of NPS (14 nmol; n = 6 rats) or saline (0.9% NaCl; n = 6) had no effect on the duration of audible vocalizations. Arthritic rats (one day postinduction) showed an increase in audible vocalizations to innocuous (Figure 1B) and noxious (Figure 1D) stimuli compared to normal rats in agreement with our previous studies [for review see [39]]. Nasal application of NPS (14 nmol; n = 8 rats), but not saline (n = 9), in arthritic rats significantly inhibited audible vocalizations to innocuous and noxious stimuli (P < 0.01 and P < 0.05, respectively; unpaired *t*-test). The data suggest that nasal application of NPS can inhibit audible vocalizations in the arthritis pain model.

#### *Ultrasonic vocalizations*

**Figure 2:**
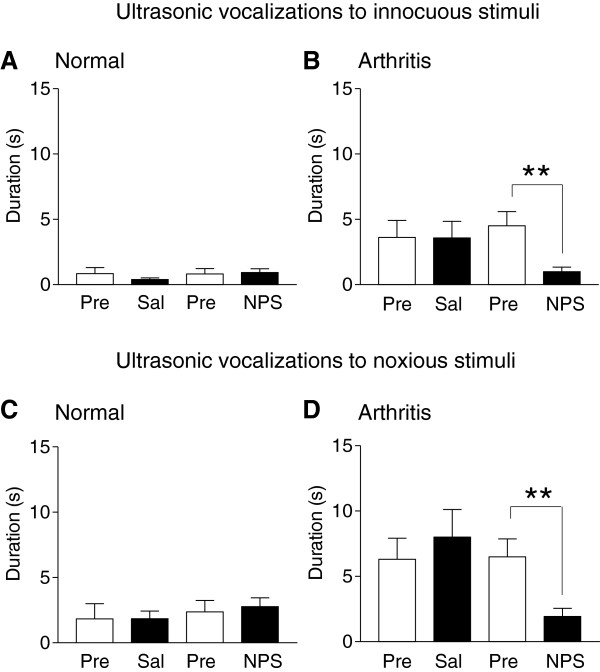
**NPS effects on ultrasonic vocalizations.** Ultrasonic vocalizations were evoked by brief (15 s) innocuous (300 g/30 mm^2^, **A**, **B**) and noxious (1200 g/30 mm^2^, **C**, **D**) stimulation of the knee joint in normal and arthritic rats. Duration of vocalizations during a 1 min period was measured before (Pre) and 45 min after nasal application of saline (Sal; n = 6 normal rats; n = 9 arthritic rats) or before (Pre) and 45 min after NPS (14 nmol; n = 6 normal rats; n = 8 arthritic rats). NPS had significant inhibitory effects in the arthritic pain model. ** P < 0.01, unpaired *t*-test.

Ultrasonic vocalizations were evoked by brief (15 s) mechanical stimuli of innocuous (300 g/30 mm^2^) and noxious (1200 g/30 mm^2^) intensities in normal (Figure 2A and C) and arthritic rats (Figure 2B and D). In normal rats, nasal application of NPS (14 nmol; n = 6 rats) or saline (0.9% NaCl; n = 6) had no effect on the duration of ultrasonic vocalizations. Arthritic rats (one day postinduction) showed increased ultrasonic vocalizations to innocuous (Figure 2B) and noxious (Figure 2D) stimuli compared to normal rats. Nasal application of NPS (14 nmol; n = 8 rats), but not saline (n = 9), in arthritic rats significantly inhibited vocalizations to innocuous and noxious stimuli (P < 0.01, unpaired *t*-test). The data suggest that nasal application of NPS can inhibit ultrasonic vocalizations of arthritic rats.

### Inhibitory effect of nasal NPS on vocalizations is reversed by an NPS receptor antagonist in the amygdala

**Figure 3:**
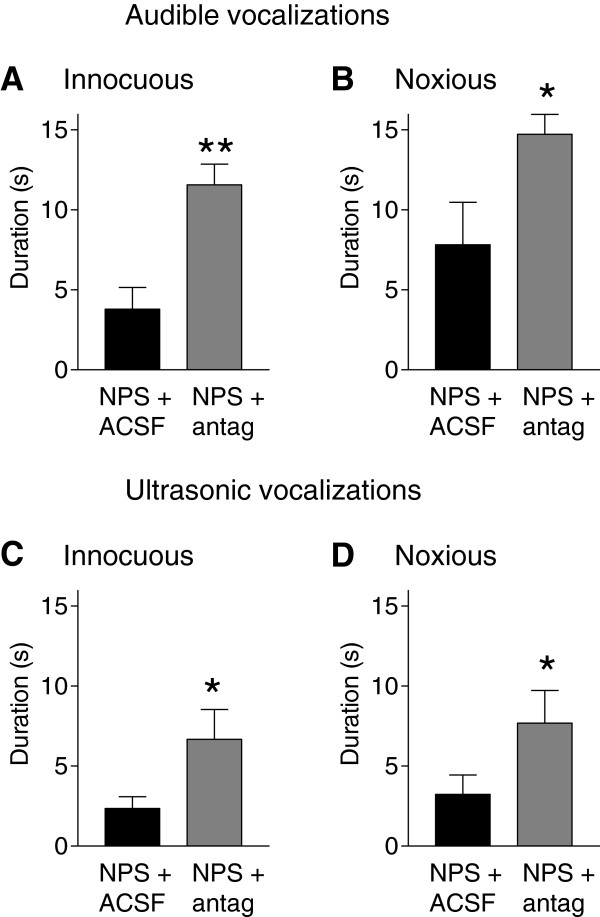
**NPSR antagonist blocks NPS effects on vocalizations.** Audible **(A)** and ultrasonic **(B)** vocalizations were evoked by brief (15 s) innocuous (300 g/30 mm^2^, **A**, **C**) and noxious (1200 g/30 mm^2^, **B**, **D**) stimulation of the knee joint in arthritic rats (4 days postinudction). Duration of vocalizations during a 1 min period was measured during stereotaxic application of an NPS receptor antagonist (SHA68, 50 μM, concentration in microdialysis probe, 20 min; n = 6 rats) or ACSF (vehicle control; n = 6 rats) following nasal application of NPS. Stereotaxic applications started 25 min after NPS and behavioral measurements were made 45 min after NPS. Compared to NPS coapplied with ACSF, vocalizations were significantly increased when NPS was coapplied with the antagonist. *,** P < 0.05, 0.01, unpaired *t*-test.

To test the hypothesis that the inhibitory effects of nasal NPS on vocalizations involve NPS receptors in the amygdala a selective NPS receptor antagonist (SHA68, 50 μM, concentration in microdialysis probe, 20 min; n = 6 rats) or ACSF (n = 6 rats) was administered into the ITC area of the amygdala 25 min after nasal application of NPS. These experiments were performed in arthritic rats 4 days postinduction. Audible (Figure 3A and B) and ultrasonic (Figure 3C and D) vocalizations of arthritic rats were significantly increased in the presence of the antagonist compared to ACSF (P < 0.05-0.01, unpaired *t*-test), suggesting that the inhibitory effect of NPS (ACSF group) was blocked by the stereotaxic application of the antagonist in the amygdala. Therefore, nasal application of NPS inhibits audible and ultrasonic vocalizations by activating NPS receptors in the amygdala. The results also show that the inhibitory effect of NPS seen in the acute stage of arthritis (day 1, Figures 1 and 2) persisted in the subacute stage (day 4, Figure 3).

### Inhibitory effects of nasal NPS on anxiety-like behavior, but not locomotion, of arthritic rats

**Figure 4:**
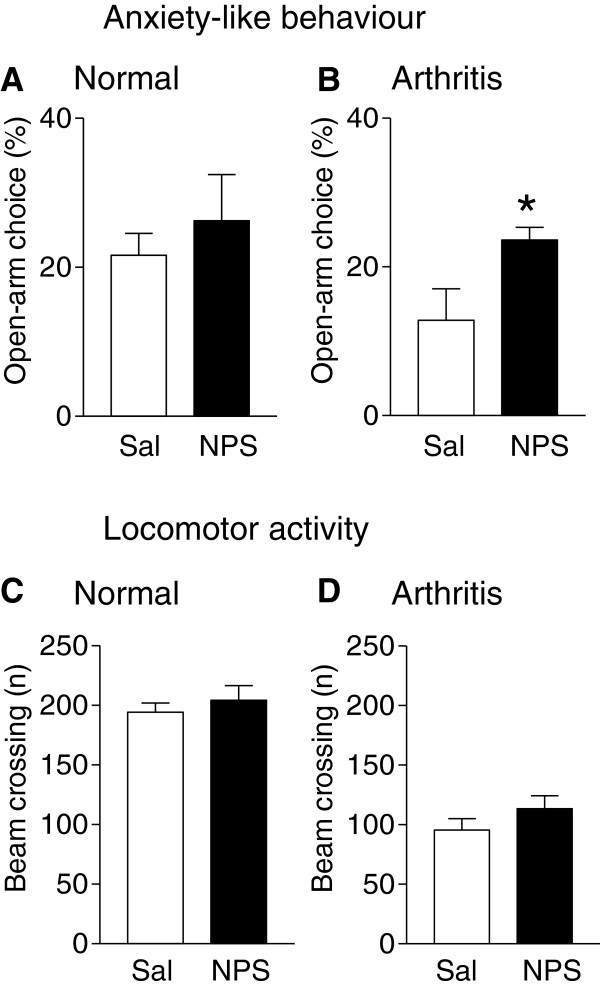
**NPS effects on anxiety-like behavior but not locomotor activity. (A, B)** Open-arm choice during the initial 5 min in the elevated plus-maze (see Methods) was measured in normal **(A)** and arthritic rats **(B)** 30 min after nasal application of saline (Sal; n = 6 normal rats; n = 6 arthritic rats) or NPS (14 nmol; n = 6 normal rats; n = 6 arthritic rats). NPS increased open-arm choice in arthritic animals significantly. * P < 0.05, unpaired *t*-test. **(C, D)** Locomotor activity was measured in the same groups of normal **(C)** and arthritic **(D)** rats as the total number of beam crossing into the 4 arms during 15 min. NPS had no significant effect (unpaired *t*-test).

Open-arm preference during the first 5 min in the elevated plus maze (EPM) was measured as an indicator of anxiety-like behavior in normal (Figure 4A) and arthritic rats (Figure 4B). Preference for the open arms was lower in arthritic rats (n = 6) than in normal rats (n = 6) as in our previous studies [29,39,40], suggesting an increase in anxiety-like behavior in the pain state. Nasal application of NPS (14 nmol) had no effect in normal rats (n = 6) but increased the open-arm choice of arthritic rats significantly (n = 6, P < 0.05, unpaired *t*-test, compared to rats treated with nasal saline, n = 6). To determine any effects of NPS on locomotor activity, the total number of beam crossing into the open and closed arms of the EPM was measured for 15 min (Figure 4C and D). Arthritic rats showed a lower number of beam crossings than normal rats, but nasal application of NPS had no significant effect on locomotor activity in normal and in arthritic rats (P > 0.05, unpaired t-tests). The data suggest that NPS can inhibit pain-related anxiety-like behavior without affecting locomotion.

### Lack of effect of nasal NPS on spinal reflexes

**Figure 5:**
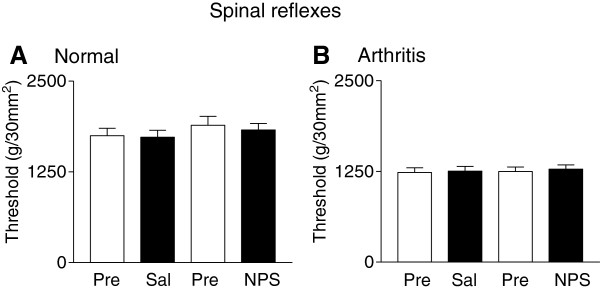
**Lack of NPS effects on hindlimb withdrawal thresholds.** Using calibrated forceps for mechanical compression of the knee joint, reflex thresholds were measured in normal **(A)** and arthritic rats **(B)** before (Pre) and 45 min after a nasal application of saline (Sal; n = 6 normal rats; n = 7 arthritic rats) or before (Pre) and 45 min after NPS (14 nmol; n = 6 normal rats; n = 7 arthritic rats). NPS had no significant effect (one-way ANOVA).

Hindlimb withdrawal thresholds were measured using mechanical compression of the knee joint with a calibrated forceps in normal (n = 6) and arthritic rats (n = 7) before and 45 min after a nasal application of saline or NPS (Figure 5). Mechanical thresholds in arthritic rats were lower than those in normal rats, but NPS had no significant effect (P > 0.05, paired *t*-test comparing predrug and drug values).

### Inhibitory effect of NPS on amygdala neurons in arthritic rats

To show directly that NPS in the amygdala inhibits pain-related neuronal activity we recorded neurons in the latero-capsular CeA, which is the output nucleus for major amygdala functions related to pain behaviors [3,4]. These experiments were performed in additional groups of animals that were not subjected to the behavioral tests. While our previous study determined the synaptic site and mechanism of action of NPS in the amygdala (ITC-driven inhibition of CeA neurons) [29], the neuronal effect of NPS on nociceptive processing in the amygdala of the intact animal has not yet been addressed. Here we recorded individual CeA neurons that responded more strongly to brief noxious than innocuous test stimuli under normal conditions, which are so-called “multireceptive” neurons according to our classification of amygdala neurons [3,4]. Test stimuli (15 s) were applied to the knee joints as in the behavioral experiments (see Methods).

All neurons (n = 17) were recorded under normal conditions; of these, 12 were also recorded continuously during the development of a subsequently induced knee joint arthritis (kaolin/carrageenan model, see Methods). Only one neuron was recorded in each rat. NPS was administered nasally or stereotaxically into the area of the ITC, which is lateral to the CeA. Our previous brain slice studies showed that NPS increased ITC-mediated synaptic inhibition of CeA neurons without acting directly on CeA or BLA neurons [29]. Based on pharmacological data from these experiment, drugs were administered by microdialysis for 20 min at a concentration 100 times that of the desired tissue concentration. Measurements of neuronal activity were made 15 min after the start of drug application.

**Figure 6:**
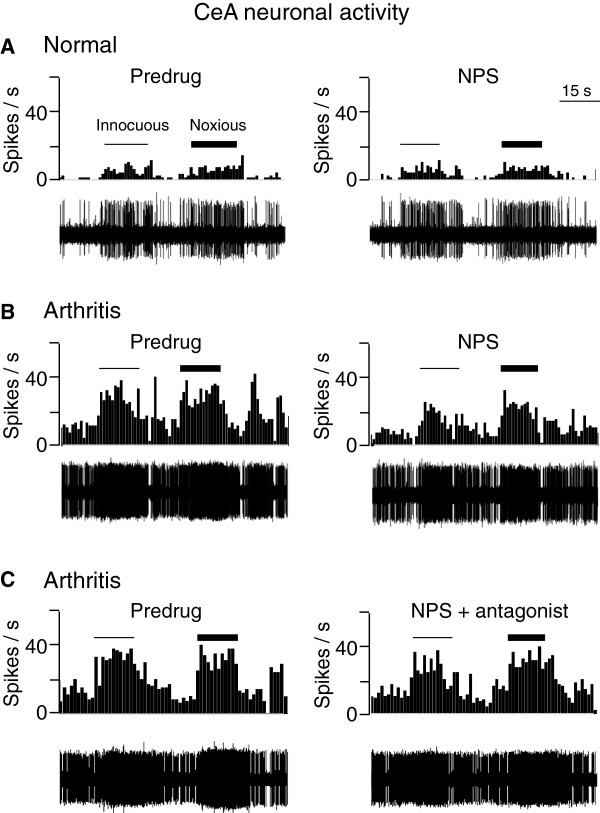
**NPS effects on individual amygdala neurons.** Extracellular single unit recordings of individual amygdala neurons in the central nucleus (CeA). Oscilloscope traces show original recordings of action potentials (spikes). Peristimulus rate histograms show spikes/s; bin width 1 s. Horizontal lines indicate innocuous and noxious test stimuli (compression of the knee joint; see Methods). **(A)** Stereotaxic administration of NPS (100 μM, concentration in microdialysis probe; 20 min) into the ITC area did not change the activity of a CeA neuron in a normal rat. **(B)** Stereotaxic administration of NPS into the ITC inhibited the activity of another CeA neuron in a rat with arthritis (6 h postinduction). **(C)** Coapplication of a selective NPSR antagonist ([D-Cys(tBu)^5^]NPS, 1 mM, concentration in microdialysis probe; 20 min) into the ITC area blocked the effect of NPS in another CeA neuron recorded in an arthritic rat (6 h postinduction).

**Figure 7:**
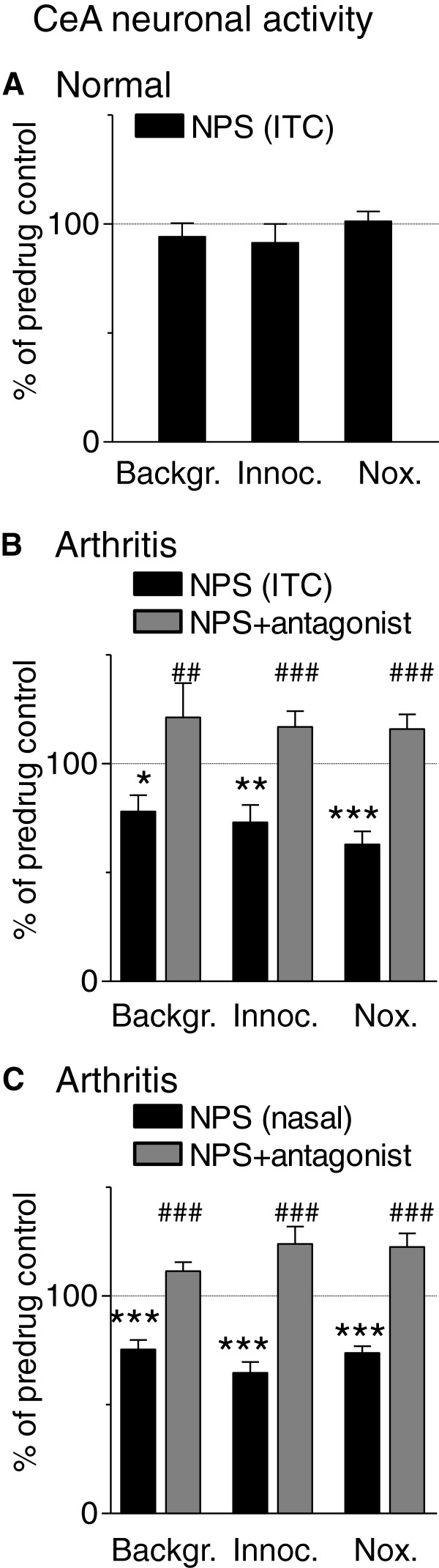
**NPS effects in the sample of amygdala neurons.** Summary of data for the sample of CeA neurons tested in normal rats (n = 5 neurons; **A**) and in arthritic rats (6-8 h postinduction; n = 20 neurons, **B** and **C**). Bar histograms show average spikes/s (mean ± SE) as percent of predrug control values (set to 100%). Statistical analysis was performed on raw data. Background activity was measured as average firing rate over 3-5 min. For evoked “net” responses, background activity (15 s) was subtracted from the total activity during stimulation (15 s). **(A)** Stereotaxic application of NPS (100 μM, concentration in microdialysis probe; 20 min) into the ITC area had no effect under normal conditions. **(B)** NPS in the ITC inhibited background and evoked activity in arthritic rats significantly (n = 12 neurons; *,**,*** P < 0.05-0.001, Newman-Keuls posttests compared to predrug). Coapplication of a selective NPSR antagonist ([D-Cys(tBu)^5^]NPS, 1 mM, concentration in microdialysis probe; 20 min) into the ITC area reversed the effect of NPS significantly (n = 7 neurons; ^##^,^###^ P < 0.01-0.001, Newman-Keuls posttests compared to NPS alone). **(C)** Nasal application of NPS inhibited background and evoked activity of CeA neurons significantly (n = 8 neurons; *** P < 0.001, Newman-Keuls posttests). Application of a selective NPSR antagonist (SHA68, 50 μM, concentration in microdialysis probe, 20 min) reversed the effect of NPS significantly (n = 6 neurons, ^###^ P < 0.001, Newman-Keuls posttests).

Figure 6 shows individual examples. Data are summarized in Figure 7. NPS (100 μM, concentration in microdialysis probe) had no effect on background or evoked activity of CeA neurons (n = 5) in normal animals without arthritis (Figures 6A and 7A). In the arthritis pain model (6-8 h postinduction), NPS significantly inhibited background and evoked activity (n = 12 neurons; P < 0.05-0.001, Newman-Keuls posttests; Figures 6B and 7B). Coapplication of a selective NPSR antagonist ([D-Cys(tBu)^5^]NPS; 1 mM, concentration in the microdialysis probe) reversed the effect of NPS significantly (n = 7 neurons; P < 0.01-0.001, Newman-Keuls posttests, Figures 6C and 7B). In another sample of CeA neurons recorded in arthritic rats, nasal application of NPS (14 nmol) also inhibited CeA activity significantly (n = 6 neurons; P < 0.001, Newman-Keuls posttests, Figure 7C) and this effect was reversed by a selective NPSR antagonist (SHA68, 50 μM, concentration in microdialysis probe, 20 min) applied stereotaxically into the ITC area (n = 6 neurons; P < 0.01-0.001, Newman-Keuls posttests). The results show that NPS acts on NPSR in the ITC area to inhibit nociceptive processing of CeA neurons in a pain state but not under normal conditions.

## Discussion

The key novelty of this is study is the finding that nasal application of recently discovered neuropeptide S (NPS) can inhibit pain behaviors in an arthritis model. This effect involves NPS receptors in the amygdala, specifically the intercalated cell (ITC) area that serves as a gate keeper of excitatory drives to amygdala output neurons in the CeA. Moreover, new electrophysiology results show that NPS administered nasally or directly into the ITC area inhibits nociceptive processing in CeA neurons that are known to play an important role in the generation and modulation of pain behaviors [3,4]. While our previous work identified ITC-driven inhibition of CeA neurons as the synaptic mechanism of action of NPS in the amygdala [29], the effect of NPS on nociceptive processing in amygdala neurons in the intact animal was not known. The behavioral and electrophysiological results are significant because the beneficial effects obtained with a non-invasive application method may suggest potential usefulness in the clinical setting, although NPS functions in humans remain to be determined. However, human NPS and NPS receptors exist and the primary sequence of NPS is highly conserved among vertebrates especially at the N-terminus with the amino acid serine (S) hence the name neuropeptide S [24,25,31].

NPS is primarily localized to discrete brainstem areas, including the lateral parabrachial nucleus and the peri-locus coeruleus [27]. These brainstem areas interact closely with the amygdala, for example through corticotropin releasing factor (CRF) expressing neurons in the lateral parabrachial nucleus and in the amygdala (CeA) [41-44]. CRF can activate NPS neurons in the locus coeruleus [45]. The amygdala CRF system plays an important role in pain modulation and generation of pain behaviors [6,46,47]. The amygdala is also one of the brain areas where NPSR is found in highest abundance [26]. NPS has been shown to control amygdala output by increasing synaptic feedforward inhibition mediated by a cluster of inhibitory ITC interneurons as a mechanism to exert anxiolytic effects [23]. Recent work from our group suggests that a similar mechanism may also account for pain-inhibiting effects of NPS [29]. Thus, NPS could be part of a homeostatic circuit of emotions involving limbic and brainstem areas [48].

In support of this notion, behavioral studies showed anxiolytic effects of NPS in the brain. Intracerebroventricular administration of NPS had anxiolytic effects in various assays and facilitated the extinction of conditioned fear responses [23,30,31,36,49]. Administration of an NPS receptor antagonist (SHA68) into the amygdala reversed the effects of NPS, suggesting a major role of the amygdala in NPS function. Intracerebroventricular administration of NPS also had antinociceptive effects in the tail-flick, hot-pate and formalin tests [34,35]. We showed previously that direct application of NPS into the ITC of the amygdala inhibited emotional-affective responses and anxiety-like behavior in an arthritis pain model but had no effect on sensory aspects (mechanical withdrawal thresholds) [29], suggesting the involvement of NPS in the amygdala in discrete aspects of pain modulation. The results of the present study provide further support of this concept.

Importantly, recent evidence suggests that NPS can be administered nasally to exert anxiolytic effects [36,37]. Topical application of NPS on the rhinarium (glabrous area around the nostrils) of rats increased novel object recognition, a common method for assessing cognitive memory enhancing effects, and had anxiolytic effects in the elevated plus-maze [36]. A study in mice reported that application of NPS to each nostril had anxiolytic effects on the elevated plus-maze and dark-light tests [37]. Locomotor activity was not affected in these studies. To the best of our knowledge, the present study is the first to examine the effects of nasal NPS on pain behaviors and amygdala activity. We also show that inhibitory effects of NPS in the pain model are mediated through NPS receptors in the amygdala, because stereotaxic application of a selective NPSR antagonist reversed the effects of NPS. Importantly, NPS had no effect under normal conditions, which is consistent with our previous study using stereotaxic application of NPS into the amygdala [29]. We interpret our data as evidence for a compensatory change in NPSR function and/or expression, which would be consistent with homeostatic functions of the NPS amygdala system. Although high levels of NPSR mRNA are found in the ITC of rats [26], NPSR protein expression appears to be absent in this area under normal conditions [27]. NPSR protein expression in the arthritis pain model remains to be determined.

Some methodological aspects need to be considered. We selected the 45 min time point after nasal NPS for the following reasons: Previous studies detected behavioral effects 30 min after nasal NPS [36,37]. Internalization of the NPS receptor-ligand complex in the brain, including the amygdala, was detected 30 min after nasal application of fluorophore-conjugated NPS [37]. In our preliminary pilot experiments, behavioral effects of nasal NPS were detected 30 min after application but became more pronounced after 45 min and were still present at 60 min. It should be noted that a previous study showed that NPS (total of 20 μl) was completely absorbed within 2 min after topical nasal application.

The dose of 14 nmol NPS was selected based on data in the literature. In rats, nasal application of NPS (4 nmol) was effective on object discrimination (memory-enhancing effect) whereas anxiolytic effects were detected with NPS (40 nmol) but not NPS (4 nmol) [36]. A study in mice tested intranasal NPS (7, 14 and 28 nmol) and found that NPS (14 nmol) was the optimum dose for anxiolytic effects [37]. We considered, but did not perform, a dose-response analysis because it would have significantly increased the number of animals without changing the key finding of this study, which is the beneficial effect of nasal NPS through an action on NPSR in the amygdala. Still, we acknowledge this as a potential shortcoming of our study.

Drug concentrations for stereotaxic application by microdialysis were derived from data in the literature and our own work [29]. In brain slices from arthritic rats, NPS (1 μM) inhibited synaptic plasticity in CeA neurons significantly through a direct action on ITC cells. In mice brain slices, NPS (10 μM) had near maximum effects on synaptic activation of ITC cells but the mechanism of action was presynaptic to ITC cells [23]. The rather indirect synaptic effect and species differences may explain differences in effective concentrations. Our previous studies comparing drug effects in brain slices and microdialysis drug application in intact animals determined that microdialysis required a 100-fold higher concentration than that needed in the tissue because of the concentration gradient across the dialysis membrane and diffusion in the tissue [29,40,46,47,50]. Drug concentrations for microdialysis application were adjusted accordingly in the present study. Importantly, NPS effects were blocked by competitive NPSR antagonists, confirming the appropriateness of the selected concentrations. We showed previously that [D-Cys(tBu)^5^]NPS, a well-established selective NPSR antagonist [51], blocked synaptic effects of NPS in the amygdala slice preparation (10 μM) and behavioral effects of NPS when applied by microdialysis (1 mM) into the ITC area [29], and so we used these concentrations in the present study. SHA68, a selective non-peptide NPSR antagonist [52,53], has become commercially available only recently. In vitro assays of calcium mobilization showed that SHA68 (10 nM to 1 μM) antagonized the effects of NPS with IC_50_ values of about 50 nM but was inactive per se [49,52]. In radioligand binding experiments a Ki value of about 50 nM was also found as well [52]. The concentration used in the microdialysis probe in our studies (50 μM) was based on the assumption that a submaximal concentration would be needed to fully antagonize the effects of NPS. This concentration is relatively low compared to those used in other studies where the injection of SHA68 (10 μM) into the amygdala antagonized behavioral effects of NPS (10 μM) and SHA68 (100 μM) antagonized the synaptic effects of NPS in mice [23]. Differences in the mode of action of NPS in the amygdala of rats (directly on ITC) and mice (indirectly, presynaptic to ITC) may explain the lower concentration needed in our study.

Finally, we did not perform placement control injections in this study. Drug injections targeted the ITC area in behavioral and electrophysiological experiments. Our previous studies on synaptic effects of NPS in brain slices showed a direct action on ITC cells but not on CeA or BLA neurons (synaptic inhibition of CeA neurons was driven by NPSR activation on ITC cells) [29]. Accordingly, administration of NPS into the CeA as a control in our previous behavioral studies had no effect [29] and therefore we did not repeat these experiments here. Although NPSR antagonist injection into the ITC area blocked the effect of nasal NPS completely, we cannot rule out the contribution of NPSR in other brain areas. For example NPSR activation by nasal NPS has been found in the ventral hippocampus [37].

## Conclusions

This study provides important new evidence that recently discovered neuropeptide S (NPS) administered using a noninvasive method (nasal application) can inhibit pain-related emotional-affective behaviors through an action involving the amygdala. Nasal application of NPS inhibited emotional-affective pain behaviors in an arthritis model. This effect involved NPS receptors in the amygdala. NPS administered nasally or directly into the intercalated cell (ITC) area inhibited nociceptive processing in CeA neurons that are known to play an important role in the generation and modulation of pain behaviors.

## Methods

Male Sprague Dawley rats (225-350 g) were housed in a temperature controlled room and maintained on a 12 h day/night cycle with continuous access to food and water. On the day of the experiment rats were transferred from the animal facility and allowed to acclimate to the laboratory for at least 1 h. After completion of the experiments, the animals were euthanized through decapitation using a guillotine (Harvard Apparatus Decapitator). All experimental procedures conform to the guidelines of the International Association for the Study of Pain (IASP) and of the National Institutes of Health (NIH) and were approved by the Institutional Animal Care and Use Committee (IACUC) at the University of Texas Medical Branch (UTMB).

### Arthritis pain model

Behavioral and electrophysiological experiments were carried out in normal and in arthritic rats. An acute arthritis was induced in the left knee joint as described before [54]. A kaolin suspension (4%, 80-100 μl) was injected into the left knee joint cavity. After repetitive flexion and extensions of the knee for 15 min, a carrageenan solution (2%, 80-100 μl) was injected into the joint cavity, and the leg was flexed and extended for another 5 min. Inflammation and swelling of the knee occur consistently after 1-3 h of induction, reach a maximum plateau at 5-6 hours and persist for several days [54].

### Behavioral tests

#### *Audible and ultrasonic vocalizations to mechanical stimulation*

Animals were briefly anesthetized with isoflurane (2%, precision vaporizer) and placed in a custom-designed recording chamber for the measurement of vocalizations. The chamber allowed access to the hind limbs for the application of mechanical (tissue compression) stimuli. Animals recovered from anesthesia and were habituated to the test environment. Audible (20 Hz to 16 Hz) and ultrasonic (25 ± 4 kHz) vocalizations were measured using a microphone and bat detector, respectively, which were placed in front of the animal at a fixed distance. Experiments were carried out in a shielded room and appropriate filtering levels were used to avoid the recording of any background noise. Brief (15 s) mechanical stimuli of innocuous (300 g/30 mm^2^) and noxious (1200 g/30 mm^2^) intensities were applied to the left knee joint, using a calibrated forceps with a force transducer to monitor the applied force (in g). Duration and number of events of audible and ultrasonic vocalizations were analyzed using Ultravox 2.0 software (Noldus Information Technology). Vocalizations were recorded for periods of 1 min starting with the onset of each mechanical stimulus.

#### *Spinal reflexes*

Thresholds of hindlimb withdrawal reflexes were measured after the vocalization tests by applying mechanical stimuli of continuously increasing intensity to the knee, using a calibrated forceps with force transducer. The area of tissue compressed by the tip of the forceps was 30 mm^2^ and the reflex threshold was expressed as g/30 mm^2^.

#### *Elevated plus-maze (EPM)*

Anxiety-like behavior was measured in normal and in arthritic rats treated with nasal NPS or saline (see Experimental protocol). The elevated plus-maze (Columbus Instruments) was used as described previously [29]. The EPM has two enclosed and two open arms that are arranged in a plus shape. The platform was elevated 70 cm above the floor. The EPM was equipped with photocells to detect animal movements in the open and closed arms. Recordings and analyses were made using Multi-Varimex software (Columbus Instruments). The animal was placed onto the central area of the plus-maze, facing an open-arm. Anxiety-like behavior was analyzed as the ratio of open-arm entries to the total number of entries (expressed as %) during 5 min. The total number of beam crossings during 15 min was used as an indicator of locomotor activity of the animal.

#### *Experimental protocol*

NPS (14 nmol) or saline (0.9% NaCl) was applied nasally 30-45 min prior to testing, because our pilot time-course data showed that nasally applied NPS had a maximum effect on vocalizations (in arthritic rats) between 30-45 min. NPS and saline were tested in normal animals and in arthritic animals. The protocol for behavioral experiments was as follows. On day 1, arthritis was induced in the arthritis group; EPM test was performed in normal animals and in arthritic animals (5-6 hours postinduction) 30 min after nasal NPS (14 nmol) or saline (0.9% NaCl). On day 2, audible and ultrasonic vocalizations were measured in normal and arthritic animals before and after nasal application of NPS (14 nmol) or saline (0.9% NaCl). On day 3, a guide cannula was implanted in arthritic animals for stereotaxic drug application (NPSR antagonist or ACSF control) by microdialysis into the ITC area of the amygdala (lateral to the central nucleus; see below). Stereotaxic injections of the antagonist or ACSF were done only in arthritic animals because NPS had no effect in normal animals. On day 4, a microdialysis probe was inserted through the guide cannula and a selective NPS receptor antagonist (SHA68, 50 μM, concentration in microdialysis probe) or ACSF (vehicle control) was applied stereotaxically into the amygdala by microdialysis 25 min after nasal application of NPS (14 nmol) to determine the ability of the antagonist to block the effects of NPS. Since NPS had effects only in arthritic rats, the antagonist was also tested only in the arthritis group. Vocalizations were measured 20 min after SHA68, i.e., 45 min after NPS.

### Electrophysiological recordings of amygdala neurons

Extracellular single-unit recordings were made from CeA neurons in rats anesthetized with pentobarbital. These animals were not tested in the behavioral experiments.

#### *Animal preparation and anesthesia*

Experimental details have been described in detail in our previous studies [40,55,56]. The animal was anesthetized with pentobarbital sodium (50 mg/kg, i.p.). Constant levels of anesthesia were maintained with pentobarbital administered through a catheter in the jugular vein (15 mg/kg per h). A cannula was inserted into the trachea for artificial respiration. After paralysis with pancuronium (0.3 mg/h, i.v.) the animal was artificially ventilated (3–3.5 ml; 55–65 strokes/min). End-tidal CO_2_ levels (kept at 4.0 ± 0.2%), heart rate and electrocardiogram (ECG) pattern were continuously monitored. Core body temperature was maintained at 37°C by means of a homeothermic blanket system. The animal was mounted in a stereotaxic frame (David Kopf Instr.) and a small unilateral craniotomy was performed at the sutura fronto-parietalis level for the insertion of the recording electrode and microdialysis probe. Electrophysiological recording and identification of CeA neurons.

Extracellular single-unit recordings were made with glass insulated carbon filament electrodes (4 - 6 MΩ) as described in detail previously [40,55,56], using the following stereotaxic coordinates [57]: 2.2-3.1 caudal to bregma, 3.8-4.2 lateral to midline, depth 6.5-8.0. The recorded signals were amplified, band-pass filtered (300 Hz to 3 kHz), displayed on an analog oscilloscope, and processed by an interface (CED 1401 Plus). Spike2 software (CED, version 4) was used for spike sorting, data storage and analysis of single-unit activity. Spike size and configuration were continuously monitored. Only those neurons were included in the study whose spike configuration matched a preset template and could be clearly discriminated from activity in the background throughout the experiment.

#### *Experimental protocol*

In each animal, background and evoked activity of only one neuron were recorded as described in detail previously [40,55,56]. Background activity in the absence of any intentional stimulation was recorded for 10 min. Brief (15 s) mechanical stimuli of innocuous (300 g/30 mm^2^) and noxious (1200 g/30 mm^2^) intensities were applied to the left knee joint as in the behavioral experiments, using a calibrated forceps with a force transducer, whose output signal was amplified, displayed in grams on an LCD screen, digitized by the CED interface, and recorded for on- and offline analysis. For the analysis of net evoked activity, background activity in the 15 s time period preceding the 15 s stimulus was subtracted from the total activity during stimulation. Neurons were selected which had a receptive field in the knee joint and responded more strongly to noxious than innocuous mechanical stimuli. After characterization of a CeA neuron, NPS was applied nasally or stereotaxically into the ITC area. In some neurons, the effect of stereotaxic application of a selective NPSR antagonist into the ITC area was tested. The antagonist was either coapplied with stereotaxically administered NPS or 25 min after nasal application of NPS as in the behavioral experiments.

### Drugs and drug application

NPS was purchased from Tocris Bioscience. SHA68, a selective non-peptide NPSR antagonist [52,53] was purchased from Bachem. [D-Cys(tBu)^5^]NPS, a well-established selective NPSR antagonist, synthesized as described before [51], was a generous gift from Drs. Remo Guerrini and Girolamo Calo, University of Ferrara, Italy.

#### *Nasal application*

NPS dissolved in saline was applied bilaterally (10 μl each) onto the glabrous skin area around the nostrils (rhinarium), but not into the nostrils, using a pipetter as described by others [36]. The animal was held in a supine position. Solutions are completely absorbed within 2 min [36].

#### *Microdialysis*

**Figure 8:**
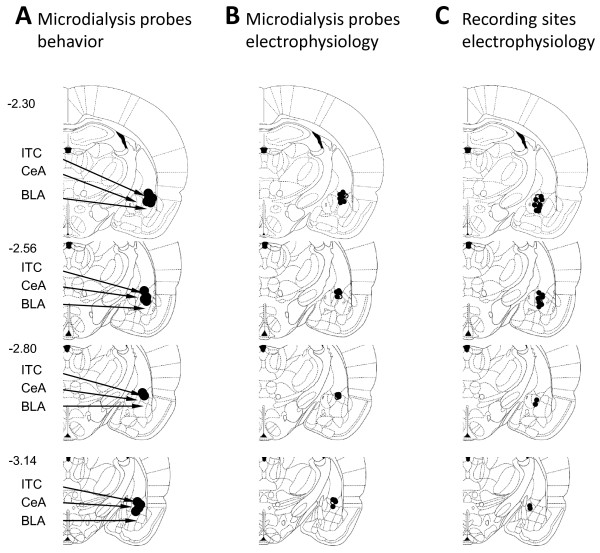
**Location of microdialysis probes and recording electrodes in the amygdala. (A)** Positions of the tips of microdialysis probes for application of an NPSR antagonist or ACSF into the ITC area of the amygdala, lateral to the CeA, in behavioral studies; n = 14. **(B)** Positions of the tips of microdialysis probes for application of NPSR antagonists into the ITC area in electrophysiology experiments; n = 5 normal, n = 18 arthritis. **(C)** Position of the tips of the glass insulated carbon filament electrodes for extracellular recordings of individual amygdala neurons in the central nucleus (CeA); n = 5 normal, n = 20, arthritis. Open circles, data from normal rats; filled circles, data from arthritic rats.

Rats for the behavioral experiments were anesthetized with pentobarbital sodium (Nembutal, 50 mg/kg, i.p.) on day 3 post-induction of arthritis. A guide canula was implanted stereotaxically into the ITC area (lateral to the CeA) of the right amygdala as described before [58], using the following coordinates [57]: 2.3-3.1 mm caudal to bregma, 4.5-4.8 mm lateral to midline, 7.2-7.6 mm depth. Guide cannulas were fastened to the skull with dental acrylic (Plastic One, Roanke, VA). The following day, a microdialysis probe (CMA/Microdialysis 11, Solona Sweden) was inserted through the guide cannula for stereotaxic drug application into the amygdala. In the electrophysiology experiments the microdialysis probe was inserted directly into the amygdala through the craniotomy (see previous section). The probe was connected to an infusion pump (Harvard Apparatus). NPS and NPSR antagonists were dissolved in artificial cerebral spinal fluid (ACSF) on the day of the experiment and applied by microdialysis at a rate of 5 μl/min. ACSF (oxygenated and equilibrated to pH 7.4) contained the following (in mM): 125.0 NaCl, 2.6 KCl, 2.5 NaH_2_PO_4_, 1.3 CaCl_2_, 0.9 MgCl_2_, 21.0 NaHCO_3_, and 3.5 glucose. The position of the microdialysis probes in the amygdala was verified histologically at the end of the experiment as in our previous studies [29] (see Figure 8).

### Statistical analysis

All averaged values are given as the mean ± standard error of the mean (SEM). GraphPad Prism 3.0 software (Graph-Pad, San Diego, CA) was used for all statistical analyses. Student’s *t*-test (paired or unpaired when appropriate) was used to compare 2 sets of data. analyses. Student’s *t*-test (paired or unpaired when appropriate) was used to compare 2 sets of data. For multiple comparisons (Figure 7), one-way analysis of variance (ANOVA) was used followed by Newman-Keuls posttests where appropriate (see Results). Statistical significance was accepted at the level P < 0.05.

## Competing interests

There are no competing interests.

## Authors’ contributions

GM and SG carried out the behavioral experiments and analyzed the data. GM created figures and provided a first draft of the manuscript. GJ performed the electrophysiology experiments and data analysis and created figures. VN conceived the study, supervised experiments and data analysis, and finalized the manuscript. All authors read and approved the final manuscript.
